# Effects of Food Properties and Aging on External Auditory Canal Movements Associated With Mastication

**DOI:** 10.7759/cureus.49475

**Published:** 2023-11-27

**Authors:** Takafumi Yamano, Kensuke Nishi, Fumitaka Omori, Takashi Kajii

**Affiliations:** 1 Section of Otorhinolaryngology, Department of Medicine, Fukuoka Dental College, Fukuoka, JPN; 2 Department of Otorhinolaryngology, Fukuoka Dental College Hospital, Fukuoka, JPN; 3 Department of Orthodontics, Keiyukai Sapporo Hospital, Sapporo, JPN

**Keywords:** food properties, aging (mri), temporomandibular joint management, muscle of mastication, external auditory ear canal

## Abstract

Few objective evaluations of external auditory canal movement during mastication have been conducted. This study investigated the extent to which age and physical properties influence such movement. The effects of food properties and aging on ear canal movement during mastication were investigated using an earable reliable chewing-count measurement device. We used such a device to study the effects of food properties and aging on ear canal movement associated with mastication.

A main effect of the difference in hardness between the foods (F = 8.3405, p = 0.0071) was found. No interaction (F = 1.3558, p = 0.2534) or main effect of age (F = 1.1206, p = 0.2982) was found. The values for peanuts were higher than those for pudding. Age had no significant effect. For both pudding and peanuts, there was a trend toward greater ear canal movement in young adults than in older adults. We suggest that external auditory canal movement during mastication decreases as muscle function declines with aging, but any effect may be less than that exerted by food properties.

## Introduction

The external auditory canal and the temporomandibular joint (TMJ) lie in close proximity, and the canal moves as the TMJ moves during mastication. Mastication is a highly coordinated neuromuscular function involving rapid and effective jaw movements and continuous coordination of various forces as the teeth bite. Activation of masticatory muscles depends on the size and texture of the food mass and facilitates the efficient grinding of food on the masticatory side [1]. One report described successful orthodontic treatment of dislocated maxillary premolars and molars of the permanent dentition, which affected mastication [2]. Objective assessment of ear canal movement may provide useful data for surgical planning of transauricular surgery and ear molding for hearing aid fitting. It may also be important for users of cerumen plugs, which are common in the elderly. However, there have been no studies on this topic to date. From a clinical perspective, it is important to collect data. An earable reliable chewing-count measurement device (RCC device) uses an optical distance sensor to measure changes in the shape of the ear canal based on changes in the relative distance between the sensor and the eardrum [3]. We used such a device to study the effects of food properties and aging on ear canal movement associated with mastication.

## Materials and methods

Participants

We enrolled 16 male volunteers without ear or TMJ disease, consisting of 8 young adults (aged 22-28 years; average age = 25 years) and 8 older adults (aged 62-67 years; average age = 64 years). Participants were invited to apply for volunteer positions from December 2022. Participants with dentures, edentulous jaws, and abnormal teeth or craniofacial morphologies were excluded. The principal investigator checked the inclusion/exclusion criteria for the participants when the participant’s first visit to the research lab.

Instruments

**Figure 1:**
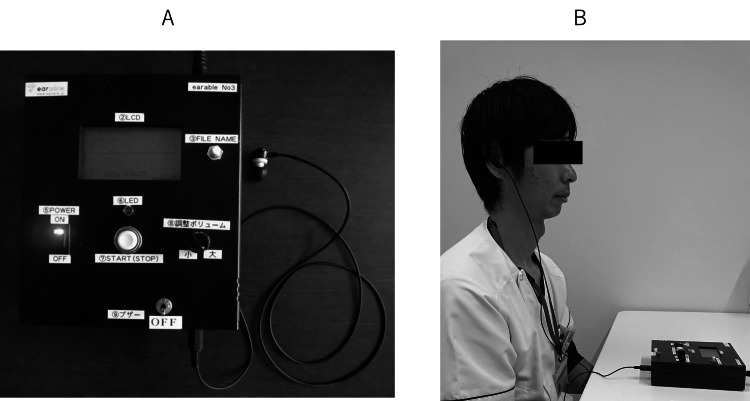
A. The earable RCC device: it has an optical distance sensor at the end of the earphone containing an infrared LED and phototransistor. B. Taking measurements RCC: reliable chewing-count

Figure 1A shows the earable RCC used in this study, which has an optical distance sensor at the end of the earphone containing an infrared LED and phototransistor. The LED emits infrared light into the ear canal, which is received by the phototransistor to measure the movement of the ear canal during mastication. The voltage waveform is displayed in real-time on a liquid crystal display in the center of the machine and the test data are recorded on a micro SD card. Figure 1B shows the actual measurement.

The advantages of this device are 1) Providing a measurement accuracy (precision and recall) of ≥90%; 2) Not impeding the patient's eating activities: less burden of wearing the device, number of chews measured without placing the sensor or device in the mouth (measurements can be taken even if there is food in the mouth), movements of the muscles and joints used for chewing (e.g., cheek joints and temporalis muscle) not obstructed by the device, and small and lightweight devices; 3) Protecting patient privacy; 4) Being easy to operate and facilitating handling without specialized knowledge; 5) Revealing the number of chews in real time to patients and physicians, recording meal contents as images, and presenting past meal contents (images) and measurements in graphical format concurrently [3].

Measurement methods

We inserted the earphone into the measuring ear and placed a mouthful of food in the subject’s mouth. The subject was instructed to chew on the same side as the earphone 30 times at a frequency of one chew/s. This was repeated for the other ear. We performed only one test daily (to avoid subject fatigue). The foods used were pudding and peanuts, which differ in hardness.

Analysis

Two-way analysis of variance (multiple comparisons (Tukey) test) was performed.

Ethical considerations

The study was approved by the Fukuoka Gakuen Research Ethics Committee (permission no. 559).

This article was previously posted to the Research Square preprint server on August 30, 2023.

## Results

**Figure 2:**
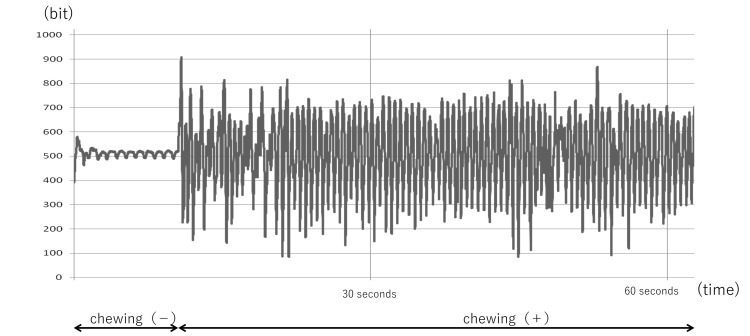
The study data The X-axis is the time direction (sampling rate = 250 times/s). The Y-axis is the direction of sensor output voltage quantized into 10 bits (1,024 steps). When the TMJ is immobile, the chewing status is set to step 512. TMJ: temporomandibular joint

The X-axis is the time direction and is sampled 250 times/s. The Y-axis is the direction of sensor output voltage quantized into 10 bits (1024 steps). When the TMJ is immobile, the chewing status is set to step 512. Changes in voltage and the ear canal shape when the mouth is opened and closed are shown in Figure 2.

The movement of the external auditory canal is represented by the relative value change of the sensor output voltage with respect to the output value 512 from the sensor when nothing is being done. In other words, the output potential difference between opening and closing is indicated with respect to the state in which the TMJ is not moving. A large amplitude means a large ear canal movement, and the graph reflects the movement of the ear canal during repeated opening and closing of the mouth.

Hence, if there is large ear canal movement associated with TMJ movement, the difference in output potential is high, as is the graphic amplitude, and vice versa. The difference in output potential multiplied by the measurement time is shown on the y-axis.

**Figure 3:**
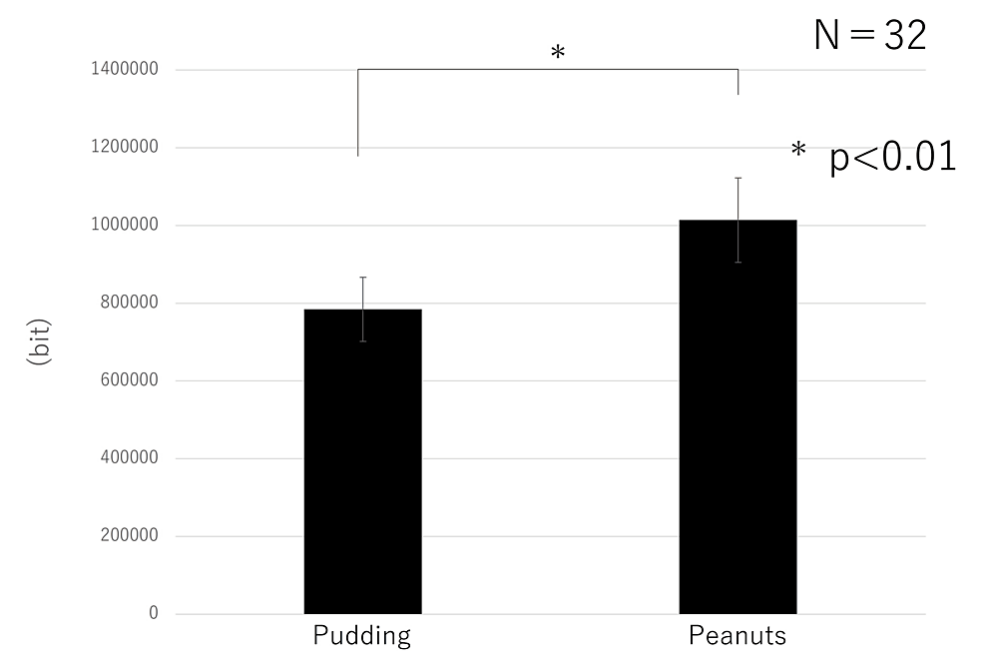
Comparison of food properties The values for peanuts were higher than those for pudding.

**Figure 4:**
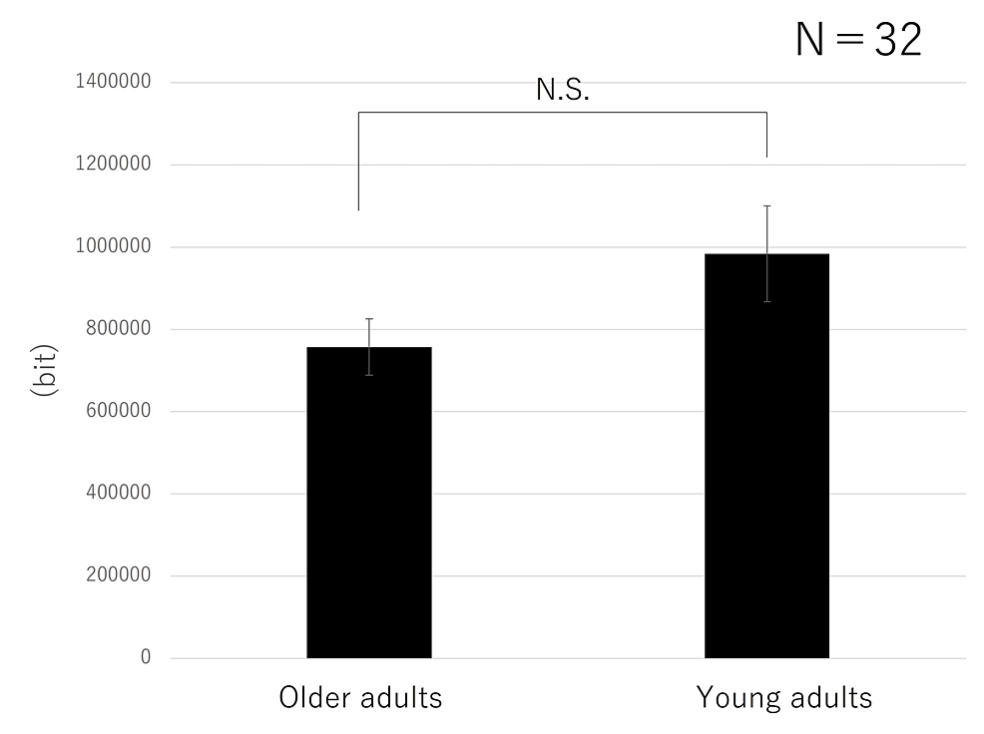
Comparison of mastication between older and young adults Age had no significant effect.

**Figure 5:**
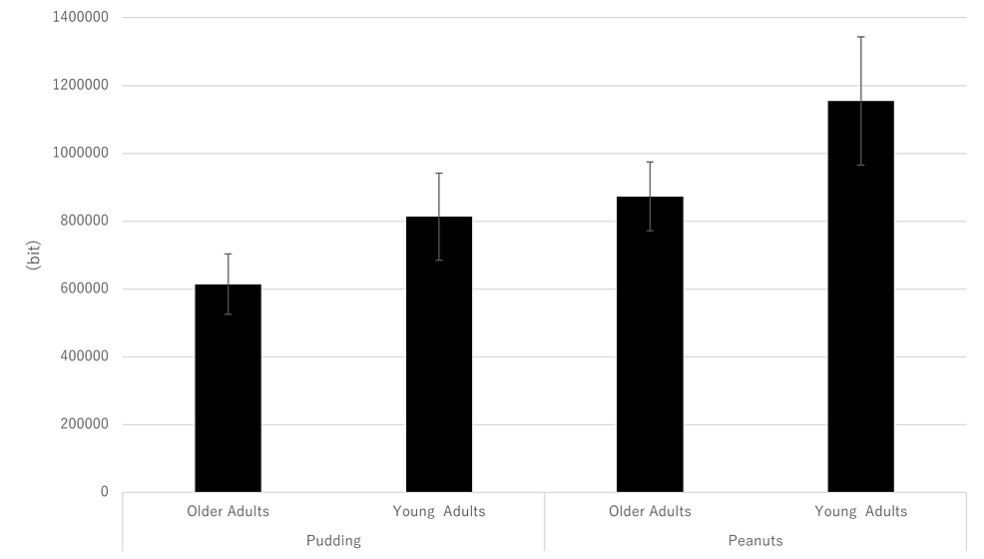
Comparison of mastication by age For both pudding and peanuts, there was a trend toward greater ear canal movement in young adults than in older adults, although the difference was not significant.

A main effect of the difference in hardness between the foods (F = 8.3405, p = 0.0071) was found. No interaction (F = 1.3558, p = 0.2534) or main effect of age (F = 1.1206, p = 0.2982) was found. The data (by food type; 30 chews) are shown in Figure 3. The values for peanuts were higher than those for pudding. Age had no significant effect (Figure 4). For both pudding and peanuts, there was a trend towards greater ear canal movement in young adults than in older adults (Figure 5). For the left and right sides, a correlation was found with a Pearson correlation coefficient of 0.433.

## Discussion

Few objective evaluations of external auditory canal movement during mastication have appeared. A few reports have explored TMJ movement by measuring the movement of the external auditory canal because jaw movement is affected by reflexes imparted by the many mechanical receptors of the teeth, gingiva, and oral mucosa. In addition, some patients cannot open the jaw. Brenman et al indirectly measured mandibular granule movement by assessing volume changes in the external auditory canal, a device that measured water pressure was attached to the canal [4]. However, this captures only a narrow range of mandibular movement and the head must bear a large device. The RCC device that we used is easy to operate, requires no special setup, and is lightweight [5]. In previous studies using this device, we reported that although there was large individuality in external auditory canal movements associated with mastication, chewing was easy on both sides [6], and the device was useful for measuring external auditory canal movements with maximum mouth opening and may aid assessment of patients with TMJ disorder [7].

One study showed that the chewing force increased as food became harder (in the order carrot > apple > banana) [8]. They used a gas pressure sensor that measured the change in ear pressure proportional to ear canal deformation during mastication. We found that the shape change of the external auditory canal was larger for peanuts than pudding and that the external auditory canal moved more when peanuts (compared to pudding) were chewed. Aging is associated with progressive loss of structure, function, coordination, and physiological integrity of the mandibular joint cartilage and subchondral bone, triggering degeneration. Electromyographic recordings of the masseter and temporalis muscles during mastication in normal subjects differed significantly by age [9-10]. The external auditory canal tended to move more in younger than in older people when consuming either pudding or peanuts, but the differences were not significant. It may be that external auditory canal movement during mastication decreases as masseter muscle function declines with age, but any effect may be less than that imparted by food properties. In addition, the present study was conducted only on adults, and we did not compare the genders. An earlier study found that mastication was influenced by craniofacial characteristics. In total, 1,071 lateral head photographs of healthy children aged 8 to 12 years with various types of occlusions were examined. The cranial maxillary index (the Se-N/PNS-A1) was greater in nine-year-old girls (p < 0.05) whereas the maxillary mandibular index (the PNS-A1/Go-Pg) was greater in nine-year-old boys (p < 0.01). Significant differences between boys and girls in terms of the length and ratio of the anterior cranial base were reported and are under further investigation [11].

In Previous reports earwax plugging was considered to be more common in the elderly and Risk factors include ear canal hairs, hearing aids, bony growths secondary to osteophyte or osteoma, and a history of impacted cerumen [12-13].

On the other hand, we hypothesize that earwax plugging reflects reduced self-cleaning of the ear canal skin, which is probably due in part to reduced motility of the ear canal. One report suggested that TMJ movement assists natural earwax expulsion [12]. In another report, 51 people aged over 65 years were categorized as ‘chewers’ (on regular or soft diets) or ‘non-chewers’ (on pureed diets or not on oral intake), ear wax deposition, and obstruction of the external auditory canal were evaluated. One report indicated that dietary habits that reduce mastication were not associated with increased cerumen in older adults [14]. However, the present study could not exclude the possibility that both food characteristics and aging may influence the movement of the external auditory canal during chewing.

The present study was conducted in a limited number of conditions, with eight healthy young adults and eight older adults, and future studies with more conditions and more cases are needed.

## Conclusions

The ear canal moved more when younger (compared to older) subjects chewed hard rather than soft food. In general, the ear canal tended to move more in younger than in older subjects, but the difference was not significant.

We suggest that external auditory canal movement during mastication decreases as muscle function declines with aging, but any effect may be less than that exerted by food properties.
